# 3D Cinema and Headache: The First Evidential Relation and Analysis of Involved Factors

**DOI:** 10.3389/fneur.2016.00030

**Published:** 2016-03-23

**Authors:** Mark Braschinsky, Aire Raidvee, Liis Sabre, Nadezhda Zmachinskaja, Olga Zukovskaja, Anti Karask, Bruno Saar, Aleksei Rakitin

**Affiliations:** ^1^Neurology Clinic, Tartu University, Tartu, Estonia; ^2^Estonian Headache Society, Tartu, Estonia; ^3^Institute of Psychology, University of Tartu, Tartu, Estonia; ^4^New York University Abu Dhabi, Abu Dhabi, United Arab Emirates; ^5^Faculty of Medicine, University of Tartu, Tartu, Estonia

**Keywords:** 3D cinema, headache, migraine, tension-type headache, chronic headache

## Abstract

**Background:**

A possible link between 3D movies and headache (HA) has never been a target of specific and systematic investigations. The aim of this study was to investigate the relationship between 3D cinema and HA and to evaluate possible risk factors of developing HA during or after watching a 3D movie.

**Methods:**

This was a prospective, non-randomized, observational study. Six thousand specifically designed questionnaires were distributed to consecutive cinema visitors. Relative HA risks for 2D- vs. 3D-movie visitors and the effects of background variables were analyzed.

**Results:**

The questionnaire was filled and returned by 1293 persons. The mean age of responders was 33.0 ± 11.3 years. Individuals who viewed 3D movies reported HA during or after the movie 1.61 times more often than 2D-movie viewers (11.1% in 3D vs. 7.2% in 2D movies, *p* = 0.017). The risk was higher in women: 2.65 times for 2D (*p* = 0.019) and 1.85 times for 3D movies (*p* = 0.06), and decreased with age by 4.6% with each year for 2D (*p* = 0.0035) and by 3.2% for 3D movies (*p* = 0.0098). Among 3D-movie visitors, those with previous HAs were 4.17 times more prone to get a cinema-induced HA (*p* = 0.02). The risk was the highest for persons with migraine (OR = 3.37, *p* = 0.001).

**Conclusion:**

For the first time, it was evidentially shown that 3D movies can provoke HA. Persons at risk are mostly younger women and/or migraineurs. Based on our results, for those belonging to the aforementioned risk groups, it can be mainly recommended to choose passive 3D technology and to view movies from the farthest possible distance.

## Introduction

Watching films in artificial 3D is a new experience for human brain. Anecdotal reports suggest a ­possible relationship between 3D movies and headache (HA). The first known attempt to draw attention to the subject was made in 2011, when Carrier asked 400 people to view the same movie in both 2D and 3D format, after which emotional reactions were measured (1). A small proportion (number not specified by the author) of participants described HA after having watched a 3D movie. Possibility of a relationship between 3D cinema and HA was mentioned in two papers by Solimini et al., where it was found that 8.3–16.8% of 3D viewers complained HA and the crude odds ratio of developing a HA after a 3D movie (vs. after a 2D movie) was calculated to be 13.16 (95% CI = 7.76–22.25) (2, 3). Read and Bohr used 3D television sets in a laboratory environment to examine possible adverse effects (4). HA was found to be one of the most frequent types of complaints in 3D groups: the probability of reporting a HA was found to vary from 2 to 10%.

Despite these few reports, the question of a possible relationship between artificial 3D experience and HA has never been a target of specific and systematic investigations.

The aim of this study was to investigate the relationship between 3D cinema and HA and to evaluate possible risk factors of developing HA during or after watching a 3D movie.

## Materials and Methods

This study was approved by and carried out in accordance with the recommendations of the Research Ethics Committee of the University of Tartu. All subjects gave written informed consent in accordance with the Declaration of Helsinki.

This was a prospective, non-randomized, observational study. Six thousand specifically designed questionnaires were proportionally distributed to consecutive cinema visitors of three major cinemas in Estonia, located in three major cities – Tallinn, Tartu, and Narva – thus, representation of major Estonian regions was ensured. The questionnaire with a prepaid reply envelope was distributed in person right before entering the cinema hall. Participants were asked to fill in the questionnaire after an exposure. Filled questionnaires were returned to the investigators via regular mail.

The questionnaire included the following information:

- demographic data,
- HA history (including previous 3D-related HA episodes),
- information about the movie theater (including location of the seat in the cinema hall) and the movie itself (including weekday and time of exposure); from the latter information, additional parameters were derived: genre of the movie, its duration, and the 3D technology (passive or active) used,
- presence of other possible HA triggers immediately before an exposure, and
- in case of an occurrence of a HA attack, several descriptors were inquired:
  ○ HA onset time and duration,
  ○ HA location,
  ○ HA characteristics,
  ○ premonitory and/or accompanying symptoms, and
  ○ treatment used, if any.

### Statistical Analysis

To investigate relative HA risks for 2D- vs. 3D-movie visitors, and the effects of background variables, ordinary logistic regression was applied using the statistical software R2.15.0 – a language and environment (5). For graphics, the R package ggplot2 was used, together with Inkscape 0.91 (6). For all analyses, statistical significance level of *α* = 0.05 was used (with two-tailed tests).

## Results

The questionnaire was filled and returned by 1293 persons (390 men and 901 women; two persons did not specify their gender), representing the response rate of 21.6%. The mean age of responders was 33.0 ± 11.3 years. There were 567 (44.2%) 3D and 715 (55.8%) 2D visits registered (not reported in 11 cases). Demographic data and basic characteristics of participants are presented in Table 1.

**Table 1:** **Characteristics of the participants**.

Total number of participants	1293
Age (mean ± SD)	33.0 ± 11.3
Gender, *n* (%)	
Male	390 (30.2)
Female	901 (69.8)
Education, *n* (%)	
Primary education	24 (1.9)
Basic education	77 (6.0)
Secondary education	264 (20.7)
Vocational secondary education	260 (20.4)
Higher education	648 (50.9)
History of HA, *n* (%)	
Yes	1115 (86.4)
No	175 (13.6)
Medical diagnosis of HA, *n* (%)	
Tension-type HA	132 (11.9)
Migraine	108 (9.7)
Trigeminal autonomic cephalalgia	6 (0.5)
Other	56 (5.0)
Unconsulted	852 (76.5)
Frequency of HA, *n* (%)	
<1 day per month	576 (52.1)
1–14 days per month	464 (42.0)
≥15 days per month	66 (6.0)
Smoking, *n* (%)	
Yes	183 (15.4)
No	1008 (84.6)

*HA, headache*.

Twenty-one persons had HA already on arrival to a ­cinema – they were excluded from the final analyses (Table 2).

**Table 2:** **HA frequency and onset time**.

Did Ha appear, *n* (%)	
Headache	133 (10.4)
No headache	1149 (89.6)
When Ha appeared, *n* (%)	
Before the movie	21 (16.4)
During the movie	60 (46.9)
After the movie	47 (36.7)

Based on the reported HA history, the baseline risk of HA was 1.4 times higher among participants in the 2D group compared to those who visited 3D cinema: 630 out of 715 (88.1%) 2D vs. 476 out of 566 (84.1%) 3D visitors had experienced HA previously, *p* = 0.038 (this analysis includes the 21 subjects who had a HA already on arrival to the cinema and were excluded from other analyses). Women were 2.66 times more likely to have previous HA history than men (*p* < 0.0001).

**Figure 1:**
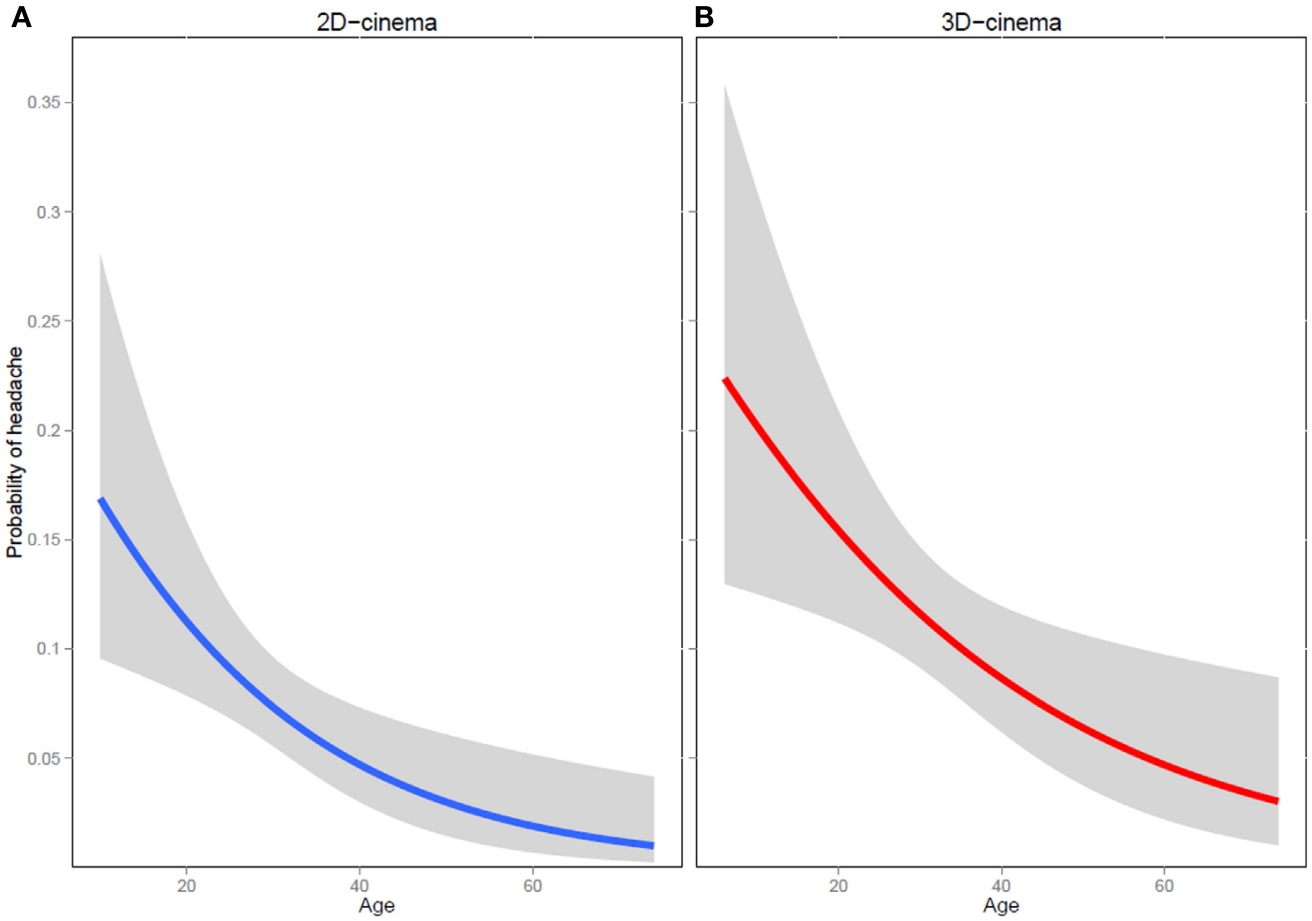
**Probability of developing a headache at different ages during or after a 2D- (A) and a 3D-movie (B)**.

Individuals who viewed 3D movies reported HA during or after the movie 1.61 times more often than 2D-movie viewers [62 out of 561 (11.1%) in 3D vs. 50 out of 697 (7.2%) in 2D movies, *p* = 0.017]. The risk was higher in women: 2.65 times for 2D (7 instances of HA out of 202 men vs. 43 instances of HA out of 495 women, *p* = 0.019) and 1.85 times for 3D movies (13 instances of HA out of 177 men vs. 49 instances of HA out of 384 women, *p* = 0.06). The risk decreased with age by a 4.6% with each year for 2D (*p* = 0.0035) and by 3.2% for 3D movies (*p* = 0.0098) (Figure 1).

The majority (86.2%) of individuals had previous HA history, yet in 2D movies previous HAs were not predictive of cinema-induced HA – the risk of getting a HA by watching a 2D movie for people with previous experience of HA compared to those without prior HAs was 1.27 (*p* = 0.62). Among 3D-movie visitors, those with previous HAs were 4.17 times more prone to get a cinema-induced HA (59 out of 411 previous HA sufferers vs. 3 out of 87 people with no previous HAs, developed a HA in 3D cinema, *p* = 0.02). In people with HA history, the risk of HA onset during or after a 3D movie was 1.81-fold compared to the same risk during/after a 2D movie (*p* = 0.004).

While in 2D movies, a history of HA in general was not predictive of cinema-induced HA, the frequency of previous HAs was relevant. Compared to people who suffered from HA less than once a month, those who had a HA for 1–14 days per month were 2.71 times and those with HAs for more than half of the days (i.e., having a chronic HA) were 6.81 times more prone to have a HA during or after a 2D movie (*p* = 0.006 and 0.0001, respectively).

In 3D movies, compared to people with HAs less than once a month, those with HA for 1–14 days per month had 1.8-fold risk and those with HAs in more than half of the days (i.e., having a chronic HA) had 5.2-fold risk of developing a HA in the movies (*p* = 0.046 and 0.0007, respectively).

**Figure 2:**
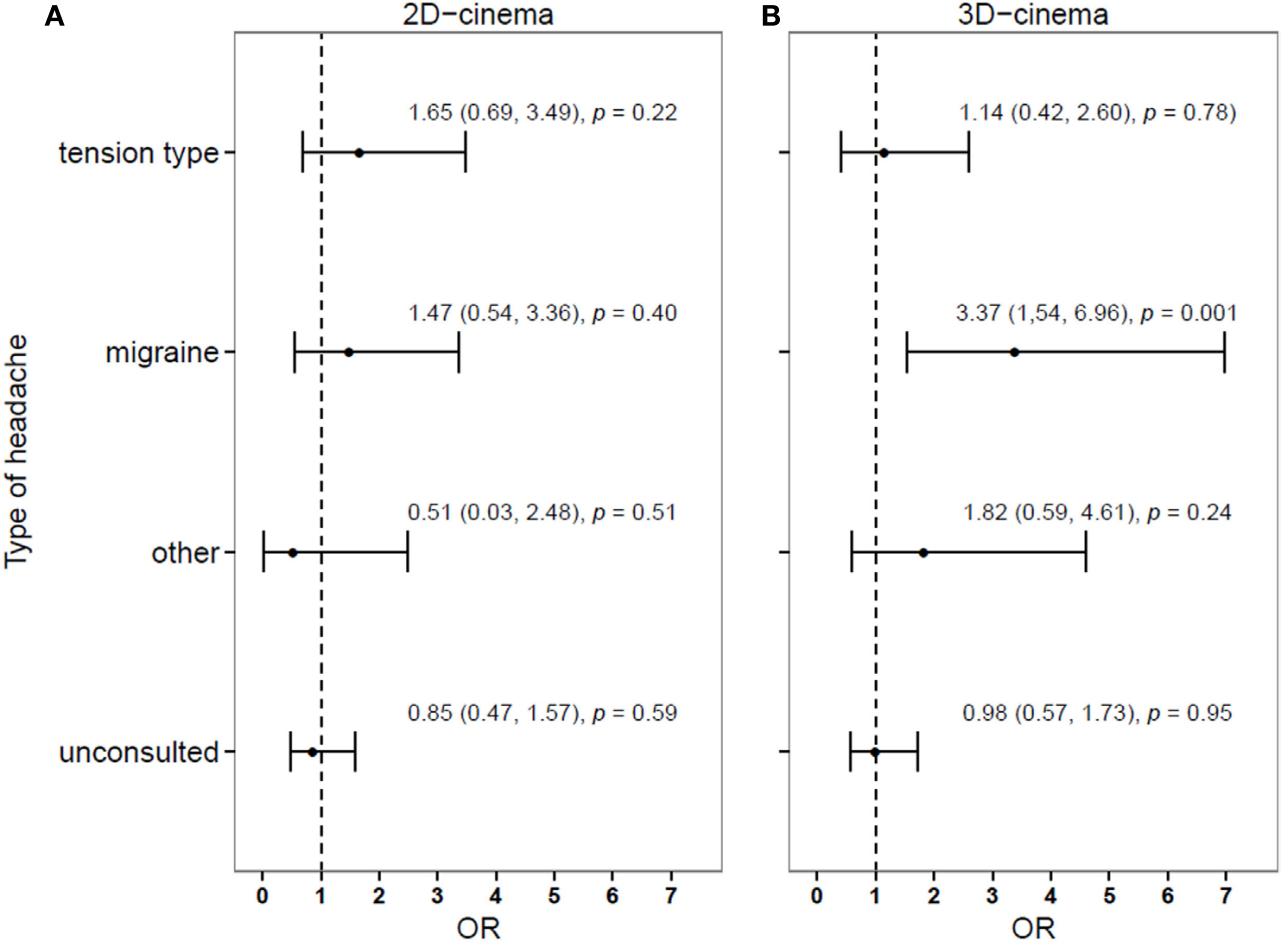
**Relative risk of developing (compared to not developing) a headache during or after a 2D- (A) or 3D-movies (B), depending on the history of headache diagnosis**.

The risk differed considerably depending on the previous diagnosis of HA, being the highest for persons with migraine (Figure 2).

**Figure 3:**
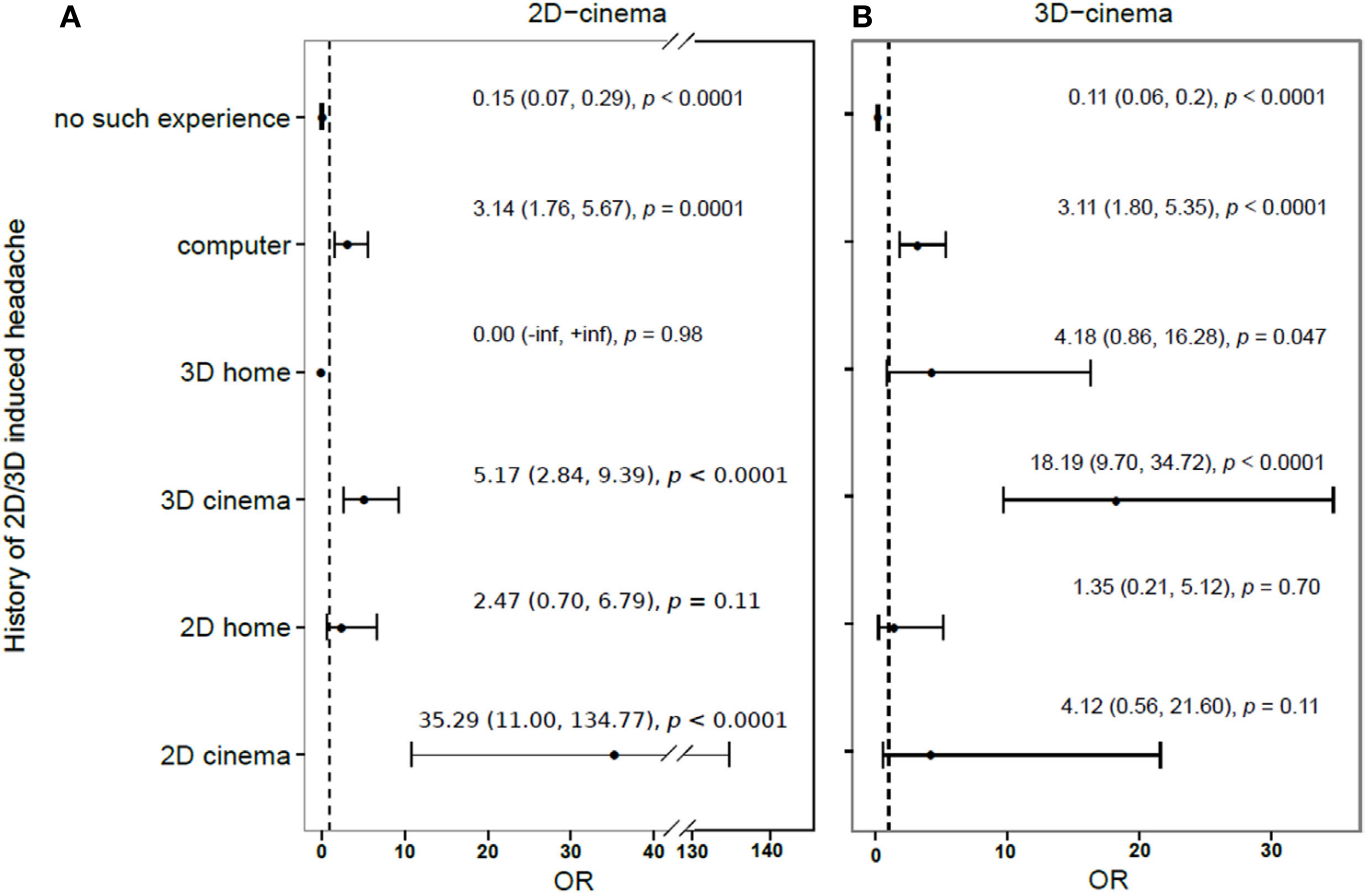
**Relative risk of developing (compared to not developing) headache during or after a 2D- (A) or 3D-movie (B), depending on the history of previous 2D/3D induced headache**.

Four hundred fifty-six out of 1267 persons (35.9%) reported having had a previous experience of getting a HA in relation to watching any 2D or 3D screens (be it at a cinema or a TV or computer screen at home). One hundred eighty six out of 1267 participants (14.7%) had had previous episodes of 3D-provoked HAs. The relation between a history of HA after an exposure to different screens and the odds of developing a HA in cinema is presented in Figure 3.

Active 3D technology was associated with 2.2 times higher probability of inducing HA than passive one, but this association was not statistically significant (*p* = 0.098).

Across 2D- and 3D-moviegoers, relationship between viewing distance from the screen at the movie theater and the probability of developing HA approached significance – seat location at the rear third of the cinema hall reduced this probability by 2.14 times when compared to the front third (*p* = 0.053). In 3D movies, compared to viewing the movie from the front third, a seat at the rear third of a cinema hall was associated with 2.1 times lower probability of HA, and at the middle third of a cinema hall with 2.9 times lower probability of HA, but none of these were statistically significant (*p* = 0.17 and 0.06, respectively). In 2D movies, the seating had no difference to the risk of developing a HA.

We found no association between HA and the genre of the movie, weekday or time of visit to the cinema. Occurrence of HA was not related to persons’ education, usage of glasses or contact lenses, visiting movie on an empty stomach, thirst, or consumption of various foods or beverages, including caffeine, alcohol, and narcotics prior to the movie.

## Discussion

To the best knowledge of the authors, this is the first study that specifically and systematically investigated a possible relationship between 3D visual experience and HA. As a general conclusion, it was evidentially shown that 3D movies can provoke HA. It was noted already at baseline, where many cinema visitors (14.7%) reported a history of developing HA after a 3D exposure. Real numbers may be somewhat different due to this study’s design – participants were all cinema visitors (not a population-based study). However, those who already had had such an experience, i.e., previous HA experience under similar conditions (either 3D movies or visiting a cinema), might well avoid visiting movies. This can be speculated and is in concordance with the analysis of HA history in our cohort: people more prone to HAs are probably more likely to avoid going to a 3D cinema. Hence, the probability of underestimation prohibits direct application of current results to the general population. However, our results are applicable to the part of general population who can be defined as “cinema visitors,” who actually represent the target group for a possibility of relation between 3D cinema and HA.

The results point out that there are some groups of persons who have higher probability of developing HA during or after viewing a 3D movie. Persons at risk are mostly younger women and/or migraineurs. According to epidemiological data, HAs are more prevalent in young adults. In general, cinema visitors tend to be younger people as well (with the risk of getting a 3D HA decreasing 3.2% for each year of age). It seems logical that there is an overlap, representing the importance of awareness for the same part of general population – young adults being not just the risk group for HAs in general but also a risk group for developing a HA during or after a 3D experience. Based on our cohort, it might seem that women, despite having a 1.85 times higher probability of 3D-provoked HAs than men, visit movies more often. But this conclusion cannot be made for several reasons. First, it was not the purpose of this study to look into gender profile of cinema visitors. Second, having a response rate of 21.6% leaves a possibility that women might be more likely to answer a questionnaire than men.

Having a history of migraine was shown to be one of the major risk factors, increasing the risk of getting a HA during or after a 3D experience approximately 3.4 times. Previous studies have looked into combined complaints, such as HA and motion sickness, after 3D movies (3). Recently published work has concluded that there is an overall increase in motion sickness susceptibility of patients with vestibular migraine (VM), but this was not different from other migraines (7). This contrasts with findings of previous studies that have shown higher susceptibility scores in questionnaires in VM than migraine in general (8).

The risk increased considerably further in relation to the frequency of HAs, being the highest for chronic HA sufferers (subjects with HA occurring on at least 15 days per month, according to the ICHD-3 beta criteria) – 5.2- to 6.8-fold increase was found in this subgroup of participants (compared to people who suffered from HA less than once a month) (9).

Although active 3D technology was related to a higher probability of developing a HA, this finding was not statistically ­significant representing just a trend in need of further investigations. Another trend found was the distance from the screen when viewing a movie: it seems that viewing a 3D film from the front third of a cinema hall represents a higher probability of developing HA.

We have looked into several other possibly related factors that showed no significant relations to the probability of HA development.

Considering the factors found to be the strongest risks for a so-called “3D HA,” it can raise a number of questions about the possible mechanisms involved. Many persons who developed a 3D-provoked HA were migraineurs. There are data that the visual system could be involved in triggering migraine attacks. Migraineurs are probably more sensitive to light stimulation that lowers pain perception thresholds (10). Light-induced discomfort and photophobia are enhanced in migraineurs, and this probably reflects an increased subcortical pain perception (11). These observations might fit well into the migraine-related hypothesis of habituation deficit but will certainly not answer the same question for other people who develop HA without having a migraine (12). Is it related to just a temporary immobilization and tension-type like pathophysiology, or can we speculate about the possibility of having a very specific and new type of HA – 3D-induced one, or are there several separate individual mechanisms in each case? These questions will have to be addressed by further studies specifically looking into the topic.

There are some limitations to this study. As it was designed to evaluate the occurrence of an event after a single exposure, it is possible neither to expand the results to general population nor to establish the repetitiveness of HAs during or after 3D films. Nevertheless, results on the relation between the history of HAs and the probability of current occurrence of 3D-provoked HA are sufficiently well-grounded. Although there was no discussion about the nature of the questionnaire with participants prior to the exposition, investigators cannot exclude the possibility that some opened the envelope and “previewed” the questionnaire that they were supposed to fill in after the movie. In other words, the design of the study did not control for an exact time of filling the questionnaire by participants. In this case, it may have influenced the onset of HA, especially for individuals who already had such experience. However, we have grounded reasons to suspect that most of participants did fill in the questionnaire as instructed – during the same or the next day. Filled questionnaires were returned via regular mail usually within 1 week after the film. In many cases, it was clear that participants started to fill in the questionnaire at least several hours after the exposure, because 36.7% of participants have reported the start of HA to be delayed after the end of the movie. The data of this study were self-reported by the participants, leaving us with a possibility of a recall-bias which could mostly affect accuracy of the history of HA diagnosis and the exact frequency of HAs. Some subgroups were too small to draw any conclusions. Unfortunately, the number of responders, who were willing to describe their HA and possible related symptoms, was very low in our study. Hence, we were not able to establish the types of cinema-induced HAs with necessary power and were not able to classify them. Last but not least, there is a possibility of a certain underestimation of some numbers due to the fact that many of those with a history of HA after a 3D experience might not visit cinema and, hence, cannot be reached by this particular study design.

Nevertheless, some practical recommendations can arise from this study. Based on our results, for those belonging to the aforementioned risk groups, it can be mainly recommended to use passive 3D technology and prefer to view movies from the farthest possible distance. Raising awareness within general population of possibilities of getting a HA during a 3D experience is warranted. It is grounded not only from a medical perspective (prophylactic measures) but also have to take into consideration the possibilities of legal consequences against cinema owners in those instances when customers might feel disappointed about not being cautioned prior to their cinema visits in terms of avoidance of negative experience while aiming for a positive one.

## Author Contributions

All authors agree to be accountable for all aspects of the work. All authors approved the final version of the manuscript. MB defined the hypothesis, designed the study, interpreted the data, drafted the manuscript, and supervised the study. AiR performed statistical analysis, interpreted the data, drafted sections of and revised the manuscript. LS interpreted the data, made significant revisions to the manuscript. NZ, OZ, AK, and BS acquired the data and revised the manuscript. AlR designed the study, interpreted the data, and made significant revisions to the manuscript.

## Conflict of Interest Statement

The authors declare that the research was conducted in the absence of any commercial or financial relationships that could be construed as a potential conflict of interest.
